# Family planning use and fertility desires among women living with HIV in Kenya

**DOI:** 10.1186/s12889-015-2218-z

**Published:** 2015-09-17

**Authors:** James Kimani, Charlotte Warren, Timothy Abuya, Richard Mutemwa, on behalf of the Integra Initiative, Susannah Mayhew, Ian Askew

**Affiliations:** Population Council, General Accident Insurance House, Ralph Bunche Road, P.O. Box 17643–00500, Nairobi, Kenya; Department of Global Health and Development, London School of Hygiene & Tropical Medicine, 15-17 Tavistock Place, WC1H 9SH London, UK

## Abstract

**Background:**

Enabling women living with HIV to effectively plan whether and when to become pregnant is an essential right; effective prevention of unintended pregnancies is also critical to reduce maternal morbidity and mortality as well as vertical transmission of HIV. The objective of this study is to examine the use of family planning (FP) services by HIV-positive and HIV-negative women in Kenya and their ability to achieve their fertility desires.

**Methods:**

Data are derived from a random sample of women seeking family planning services in public health facilities in Kenya who had declared their HIV status (1887 at baseline and 1224 at endline) and who participated in a longitudinal study (the INTEGRA Initiative) that measured the benefits/costs of integrating HIV and sexual/reproductive health services in public health facilities. The dependent variables were FP use in the last 12 months and fertility desires (whether a woman wants more children or not). The key independent variable was HIV status (positive and negative). Descriptive statistics and multivariate logistic regression analysis were used to describe the women’s characteristics and to examine the relationship between FP use, fertility desires and HIV status.

**Results:**

At baseline, 13 % of the women sampled were HIV-positive. A slightly higher proportion of HIV-positive women were significantly associated with the use of FP in the last 12 months and dual use of FP compared to HIV-negative women. Regardless of HIV status, short-acting contraceptives were the most commonly used FP methods. A higher proportion of HIV-positive women were more likely to be associated with unintended (both mistimed and unwanted) pregnancies and a desire not to have more children. After adjusting for confounding factors, the multivariate results showed that HIV-positive women were significantly more likely to be associated with dual use of FP (OR = 3.2; *p* < 0.05). Type of health facility, marital status and household wealth status were factors associated with FP use. Factors associated with fertility desires were age, education level and household wealth status.

**Conclusions:**

The findings highlight important gaps related to utilization of FP among WLHIV. Despite having a greater likelihood of reported use of FP, HIV-positive women were more likely to have had an unintended pregnancy compared to HIV-negative women. This calls for need to strengthen family planning services for WLHIV to ensure they have better access to a wide range of FP methods. There is need to encourage the use of long-acting reversible contraceptive (LARC) to reduce the risk of unintended pregnancy and prevention of vertical transmission of HIV. However, such policies should be based on respect for women’s right to informed reproductive choice in the context of HIV/AIDS.

**Trial registration:**

NCT01694862

## Background

According to the UNAIDS, at the end of 2010, an estimated 34 million people were living with HIV and sub-Saharan Africa continues to be disproportionately affected by the epidemic. The region accounts for about 68 % of all people living with HIV with women accounting for over half (59 %) of all people living with HIV globally [1]. With major efforts directed at expanding access to life-saving antiretroviral therapy (ART) in sub-Saharan Africa [2], many people on ART are leading productive and sexually active lives and so face challenges in having pregnancies only when intended. Unintended pregnancies (this includes both unwanted and mistimed) and the potential of vertical transmission of HIV to the child are some of the challenges faced by HIV-positive women. Unintended pregnancies for all women can have negative health, economic and social consequences for the woman and child, including increased maternal morbidity and mortality, poor breastfeeding and nutritional status and infant mortality [3]. For HIV-positive women, the likelihood of adverse health outcomes associated with pregnancy are elevated due to a number of factors including faster decline in CD4 count after pregnancy, HIV-related infections and co-morbid conditions (for example, diabetes) [4–9].

Studies in Kenya and Malawi found that 54 and 40 % of HIV-positive women, respectively, reported that their last childbirth was unintended and modern contraceptive use was lower among HIV-positive than HIV-negative women (26 vs. 46 % in Kenya; 19 vs. 46 % in Malawi). Among HIV-positive women who did not want a child ever or in the next two years, less than a third in Kenya and less than a fifth in Malawi were using a modern contraceptive [10].

Enabling women living with HIV to use contraception effectively is a cost-effective strategy that can decrease the number of unintended pregnancies and in turn reduce maternal mortality and vertical transmission of HIV [11–17]. However, this strategy is undervalued and not fully appreciated by those responsible for implementing SRH and HIV programs [18]. At the global level, prevention of unintended pregnancies among women living with HIV has been identified as one of the major components of a comprehensive strategy on prevention of mother-to-child (PMTCT) HIV transmission [19].

Women living with HIV, like all women, have a right to make and act freely and voluntarily upon their reproductive decisions, including whether and when to become pregnant. The ability to bear children continues to have a high social value in Kenya and many women living with HIV desire to become pregnant to avoid being shunned or stigmatized or to sustain their marriage and to leave children as their legacy should they die from AIDS [20, 21]. Understanding the fertility preferences and reproductive decisions of HIV-positive women is vital for informing policy and programmatic efforts to enable them to achieve these desires effectively and safely). Evidence on fertility desires and family planning among WLHIV has been growing [22–32]. This study describes and compares use of family planning and fertility desires among HIV-positive and HIV-negative women attending 12 rural and peri-urban public health facilities for FP services in five counties in the Central region of Kenya.

## Methods

### Description of intervention

The Integra Initiative was a multi-country research study measuring the feasibility, effects and costs of integrated HIV and sexual and reproductive health services in Kenya and Swaziland. The integrated HIV and FP service model developed explicit linkages with FP services and relevant HIV/AIDS services, and enabling linkage with antiretroviral therapy (ART) services for eligible clients, either on-site or through referral to other health facilities. Before recruitment of participants, providers in study intervention facilities were trained in provision of integrated services using a Balanced Counselling Strategy Plus algorithm (BCS+) and a standardised mentorship strategy described elsewhere [33]. Study implementation begun after intervention-facility providers were certified as attaining a pre-determined minimum level of clinical skills. To be eligible for inclusion in the FP-HIV study, the women had to be aged 15 years and over, be revisit FP clients, be living in the catchment area of the health facility, and willing to give their informed consent to be interviewed. The study methodology used to evaluate the intervention is described in detail elsewhere [34].

### Study population and sampling

The data were collected through the INTEGRA Initiative - a multi-country research study measuring the benefits and costs of integrated HIV and sexual and reproductive health services (www.integrainitiative.org). The study sample was selected randomly from women seeking family planning services at 12 public health facilities in Kenya. Participants were recruited between November 2009 and May 2010 as a cohort of women using FP that were followed for 24 months during which measures were made three times to determine trends in several reproductive health indicators, including fertility desires, pregnancy status (both planned and unintended), consistency in use of FP, and HIV status.

A total of 1959 women were recruited at baseline, of which 1636 (83.5 %) were reported being HIV-negative and 251 (12.8 %) reported being HIV-positive; 72 women declined to report their HIV status. At endline, 1224 women remained in the cohort, of which 1068 (87.3 %) were HIV-negative and 156 (12.7 %) were HIV-positive. For this analysis, we used baseline and endline data only, and included only women willing to report their HIV status. The desired sample size of 1959 was calculated to test the larger study hypothesis that exposure to the FP model of intervention would lead to an increase in condom use in addition to another contraceptive method by at least 5 percent among sexually active women over two years and to allow for 30 % loss-to-follow up.

The women were recruited from 12 public health facilities which were purposively selected based on provision of a minimum range of services (FP, voluntary counseling and testing (VCT), STI treatment, and PMTCT) and a minimum number of FP clients (100 or more per month). The health facilities were located in peri-urban and rural areas of five counties in Kenya; the facilities comprised four hospitals and eight health centres. All women seeking FP services from the health facilities on the days when the research team was present were approached for recruitment until the desired sample size was reached. For inclusion, the women had to be aged 15 years and over, live in the catchment area of the health facility, and give their informed consent to be interviewed. All adolescents 15 – 17 years were only interviewed following parental consent.

### Data collection

A closed-ended questionnaire was used to collect data on women’s fertility intentions, pregnancy, use of FP, other SRH and STI/HIV-related behaviors and health-seeking behaviors. Women were also asked whether they ever had an HIV test, whether they knew their status, and if so, whether they were willing to voluntarily disclose their status. There was no pressure for them to disclose their HIV status and unwillingness to do so was not a criterion for exclusion from participating in the INTEGRA study. Trained research assistants conducted the interviews using hand-held personal digital assistants (PDAs) loaded with the questionnaire tool translated into Swahili. Every respondent was given a full description of the study and gave their informed consent in writing prior to interview.

### Data analysis

Descriptive, bivariate and multivariate analyses were carried out using STATA ® version 10. The descriptive and bivariate analyses were used to describe the characteristics of the sample and explore the associations between FP use in the last 12 months, fertility desires and the woman’s HIV status. Chi-square (*X*^2^) and Fisher’s Exact tests were used for bivariate analyses. Multivariate logistic regression analysis was conducted to examine these relationships controlling for potential confounding factors (including marital status, education, and household socioeconomic status (SES) identified in previous research [21, 30, 35–37]. The analysis models included an interaction term between HIV status and timing of data collection to assess changes in FP use and fertility desires over time.

Table 1 summarizes the operational definitions for the study variables.

**Table 1:** Definition of variables in the analysis

Variable	Operational definition
Dependent variables	
Used family planning method in last 12 months	Yes = 1 and No = 0
Desires more children	Yes = 1, No = 0 and Undecided = 2
Key independent variables	
HIV status	Positive = 1 and Negative = 0
Number of children desired	Average number desired
Facility type	Health center = 1 and Hospital = 0
Dual protection in the last 12 months (condom use with another FP method)	Yes = 1 and No = 0
Timing of next birth	1 to 2 years = 1 and 3 years or more = 2
Intention regarding last birth	Wanted to be pregnant = 1, Mistimed = 2, and Unwanted = 3
Other independent variables	
Age of respondents	0 = 15-24 years, 1 = 25-34 years, 2 = 35+ years
Marital status	0 = Married, 1 = Single, and 2 = Divorced/separated/widowed
Education	0 = Primary and below, 1 = Secondary/higher
Household SES^a^	0 = Non-poor and 1 = Poor

^a^Household SES was computed using the principal component analysis technique and the items used for computation included ownership of different household items such as television, radio, bicycle and use of different types of sources of fuel for cooking

### Ethical considerations

The study was approved by the Kenya Medical Research Institute (KEMRI) Ethical Review Board (IRB approval numbers 113 and 114), the Population Council’s Institutional Review Board (IRB approval numbers 443 and 444), and the Ethics Review Committee of the London School of Hygiene & Tropical Medicine (LSHTM) (IRB approval number 5426). The Integra Initiative is registered on the Clinical Trials registration site: ClinicalTrials.gov Identifier: NCT01694862. All research staff were trained and certified in research ethics. Written informed consent was obtained from all participants.

## Results

### Descriptive analysis

Table 2 summarizes the socio-demographic characteristics of the study samples at baseline. HIV-positive women were older, much less likely to be married, were poorer and less educated. Most HIV-positive women (94 %) and 66 % of HIV-negative women visited hospitals to seek for care.

**Table 2:** Socio-demographic characteristics by HIV status at baseline

	HIV-positive (*N* = 251)	HIV-negative (*N* = 1,636)	*p* value
	%	%	
Age of respondents			
15–24 years	4	32	<0.001
25–34 years	51	51	
35+ years	45	17	
Mean age in years (SD)	34 (6.1)	28 (6.0)	<0.001
Marital status			
Single	22	3	<0.001
Married	62	96	
Divorced/separated/widowed	16	1	
Education			
Primary and below	71	58	<0.001
Secondary and higher	29	42	
Household SES			
Poor	56	47	0.020
Non-poor	44	53	

Table 3 presents the bivariate associations between HIV status and explanatory variables at baseline and endline. Overall, the sample size at endline is smaller than at baseline and this could be attributed to loss-to-follow up. For example, among HIV-positive women, death from AIDS complications or migration could be plausible explanations.

**Table 3:** Bivariate associations between HIV status and explanatory variables

	Baseline			Endline		
	HIV-positive	HIV-negative		HIV-positive	HIV-negative	
	% (N)	% (N)	*p*-values	% (N)	% (N)	*p*-values
Desire more children						
No	80 (201)	44 (727)		82 (66)	57 (300)	***
Yes	18 (46)	52 (843)	***	15 (12)	42 (218)	
Undecided	2 (4)	4 (65)		3 (2)	1 (6)	
Average number of children desired (standard deviation)	3.0 (1.4)	3.1 (1.0)	0.323	3.2 (1.1)	3.3 (1.0)	0.278
Timing of next birth						
1–2 years	52 (24)	20 (169)		36 (5)	29 (64)	0.568
3 years or more	48 (22)	80 (674)	***	64 (9)	71 (160)	
Intention regarding last birth						
Wanted to be pregnant then	57 (142)	70 (1,144)		33 (4)	71 (72)	**
Mistimed (wanted to be pregnant later)	28 (70)	21 (347)	***	25 (3)	24 (24)	
Unwanted (wanted no more children)	15 (38)	9 (142)		42 (5)	5 (5)	
Used family planning in last 12 months						0.772
No	1 (3)	7 (121)	***	10 (16)	10 (102)	
Yes	99 (247)	93 (1,505)		90 (140)	91 (966)	
Dual protection in last 12 months						
No	59 (146)	97 (1,461)	***	13 (14)	92 (879)	***
Yes	41 (101)	3 (44)		87 (90)	8 (72)	
Facility type						
Hospital	94 (237)	67 (1,105)		96 (150)	67 (721)	***
Health center	6 (14)	33 (531)	***	4 (6)	33 (347)	
Age of respondents						
15–24 years	4 (11)	32 (526)	***	1 (1)	15 (161)	***
25–34 years	51 (128)	51 (838)		44 (69)	56 (598)	
35+ years	45 (112)	17 (272)		55 (86)	29 (309)	
Marital status						
Single	22 (56)	3 (45)		21 (33)	4 (38)	***
Married	62 (155)	96 (1,572)	***	57 (89)	93 (994)	
Divorced/separated/widowed	16 (40)	1 (19)		22 (34)	3 (36)	
Education						
Primary and below	71 (178)	58 (955)	**	71 (108)	58(612)	**
Secondary and higher	29 (73)	42 (681)		29 (43)	42 (434)	
Household SES						
Poor	56 (140)	47 (771)	0.011	56 (88)	51 (546)	0.217
Non-poor	44 (111)	53 (865)		44 (68)	49 (522)	

*p < 0.05; **p < 0.01; ***p < 0.001; *X* ^2^ and Fisher’s Exact test were used to test the association between HIV status and explanatory variables

#### Fertility desires and HIV status

Significantly higher proportions of HIV-positive women reported not wanting to have more children but there were no significant differences in the number of children desired (Table 3). Further analysis showed that more HIV-positive women than HIV-negative women already had their desired number of children (60 vs. 39 %; *p* < 0.001). At baseline, HIV-positive women were more likely to report wanting to become pregnant within one to two years (52 vs. 20 %; *p* < 0.001) than HIV-negative women, but at endline there was no statistically significant difference.

At baseline, a significantly higher proportion of HIV-positive women reported that their last birth was unintended (either mistimed or unwanted). At endline, only 12 HIV-positive women had a pregnancy since recruitment and out of these, eight said their pregnancy was unintended.

#### Family planning use and HIV status

At baseline, slightly more HIV-positive women than HIV-negative women used family planning in the last 12 months (*p* < 0.001). At endline, the proportion of those who used family planning decreased in both groups but the margin was higher among HIV-positive women (*p* = 0.772)(Table 3). Short-term FP methods were the mostly commonly used methods in the last 12 months (Table 4). In both study rounds, fewer HIV-positive than HIV-negative women used hormonal pills and injectables; however, there was an increase in the proportion of HIV-positive women who used the two FP methods. More HIV-positive than HIV-negative women used male condoms at baseline (41 vs. 1 %; *p* < 0.001) and endline (26 vs. 2 %; *p* < 0.001). Women who were recruited at health centers (regardless of HIV status) were more likely to use hormonal pills than women recruited at hospitals (37 vs. 26 %; *p* < 0.001). There were no significant differences by facility type among women who used injectables (53 vs. 51 %; *p* = 0.254). More women who were recruited at hospitals (regardless of HIV status) reported using implants and IUCDs than women recruited at health centers (6 vs. 2 %; *p* < 0.001) and (8 vs. 5 %; *p* = 0.006), respectively.

**Table 4:** Type of FP methods used in the last 12 months by HIV status

	Baseline			Endline		
	HIV-positive (*N* = 247)	HIV-negative (*N* = 1,505)	*p* value	HIV-positive (*N* = 140)	HIV-negative (*N* = 966)	*p* value
	% (n)	% (n)		% (n)	% (n)	
Hormonal pills	13 (32)	32 (485)	<0.001	14 (19)	30 (294)	<0.001
Injectables	36 (90)	59 (880)	<0.001	41 (57)	45 (433)	0.360
Male condoms	41 (101)	1 (18)	<0.001	26 (36)	2 (15)	<0.001
IUCD	3 (8)	4 (55)	0.745	6 (9)	15 (140)	0.009
Implants	2 (6)	3 (51)	0.431	11 (15)	7 (67)	0.111
Female sterilization	2 (6)	0 (0)	<0.001	3 (4)	1 (8)	0.030

Further analysis showed that among women with unintended pregnancy (regardless of HIV status), a majority reported using injectables (52 %), 25 % used hormonal pills, 8 % used IUCDs and 6 % used implants.

#### Dual protection and HIV status

Dual protection (use of condom and another FP method) was significantly higher among HIV-positive than HIV-negative women at baseline (41 vs. 3 %; *p* < 0.001) and endline (87 vs. 8 %; *p* < 0.001), and increased dramatically over the two-year period; among HIV-negative women there was also a significant increase in the proportion reporting dual protection (Table 3).

### Multivariate analysis

#### Family planning use and HIV status

Table 5 presents results from the multivariate regression analysis. At baseline, HIV-positive women were 8.5 times more likely to have used FP during the previous 12 months than HIV-negative women. Contraceptive use during the previous 12 months declined among both groups at endline, but the decline was higher among HIV-positive women.

**Table 5:** Adjusted odds ratios (ORs) and 95 % confidence intervals (CIs) for family planning use and explanatory variables

Variables	Family planning use			
	Without interaction term (HIV status and study round)		With interaction term (HIV status and study round)	
	Adjusted OR	95 % CI	Adjusted OR	95 % CI
HIV status (Ref = HIV-negative)				
HIV-positive	3.4**	[1.7–6.8]	8.5**	[2.4–30.5]
Study round (Ref = Baseline)				
Endline	0.7*	[0.5–1.0]	0.8	[0.6-1.2]
Facility type (Ref = Hospital)				
Health center	1.5*	[1.0–2.2]	1.5*	[1.0-2.1]
Desire more children (Ref = Yes/undecided)				
No	0.9	[0.6–1.4]	0.9	[0.6–1.4]
Number of children desired	0.9	[0.8–1.1]	0.9	[0.8–1.1]
Age of respondents (Ref = 15-24 years)				
25-34 years	1.0	[0.7–1.5]	1.0	[0.7–1.4]
35+ years	1.3	[0.7–2.3]	1.3	[0.7–2.3]
Marital status (Ref = Married)				
Not married	0.5*	[0.3–0.9]	0.5*	[0.3–0.9]
Education (Ref = Primary and below)				
Secondary and higher	0.8	[0.6–1.1]	0.8	[0.6–1.2]
Household SES (Ref = Non-poor)				
Poor	1.5*	[1.1–2.2]	1.5*	[1.1–2.2]

*p < 0.05; **p < 0.01; ***p < 0.001; Ref = reference category

Women who were recruited at health centers were significantly more likely to have used FP in the previous 12 months than women recruited at hospitals. Unmarried and non-poor women, regardless of HIV status, were less likely to have used FP in the previous 12 months; education did not influence FP use.

#### Dual protection and HIV status

As indicated in Table 6, HIV-positive women were 17 times more likely to use dual protection than HIV-negative women and this increased substantially over time. The only other influential factor was marital status, with unmarried women more than two and a half times more likely to practice dual protection than married women.

**Table 6:** Adjusted odds ratios (ORs) and 95 % confidence intervals (CIs) for dual use of family planning and explanatory variables

Variables	Dual use of family planning			
	Without interaction term (HIV status and study round)		With interaction term (HIV status and study round)	
	Adjusted OR	95 % CI	Adjusted OR	95 % CI
HIV status (Ref = HIV-negative)				
HIV-positive	22.8***	[13.8–37.8]	17.0***	[9.9–29.3]
Study round (Ref = Baseline)				
Endline	3.3***	[2.3–4.8]	2.2**	[1.4-3.6]
Facility type (Ref = Hospital)				
Health center	0.8	[0.5–1.2]	0.8	[0.5-1.2]
Desire more children (Ref = Yes/undecided)				
No	1.1	[0.8–1.7]	1.2	[0.8–1.8]
Number of children desired	1.0	[0.8–1.1]	1.0	[0.8–1.2]
Age of respondents (Ref = 15-24 years)				
25-34 years	1.2	[0.7–1.9]	1.2	[0.7-2.0]
35+ years	0.6	[0.3–1.2]	0.7	[0.3–1.3]
Marital status (Ref = Married)				
Not married	2.4**	[1.4–4.1]	2.6**	[1.5–4.4]
Education (Ref = Primary and below)				
Secondary and higher	1.1	[0.7–1.6]	1.1	[0.7–1.6]
Household SES (Ref = Non-poor)				
Poor	1.0	[0.7–1.5]	1.0	[0.7–1.5]

*p < 0.05; **p < 0.01; ***p < 0.001; Ref = reference category

#### Fertility desires and HIV status

Table 7 shows that HIV-positive women were 2.5 times more likely not to want more children than HIV-negative women. Regardless of HIV status, older, poorer, less educated women were more likely to want no more children; marital status was not influential.

**Table 7:** Adjusted odds ratios (ORs) and 95 % confidence intervals (CIs) for fertility desires (wanting no more children) and explanatory variables

Variables	Fertility desires (wanting no more children)			
	Without interaction term (HIV status and study round)		With interaction term (HIV status and study round)	
	Adjusted OR	95 % CI	Adjusted OR	95 % CI
HIV status (Ref = HIV-negative)				
HIV-positive	2.3***	[1.6–3.4]	2.5***	[1.6–3.7]
Study round (Ref = Baseline)				
Endline	1.0	[0.8–1.2]	1.0	[0.8-1.2]
Facility type (Ref = Hospital)				
Health center	1.0	[0.8–1.3]	1.0	[0.8-1.3]
Age of respondents (Ref = 15–24 years)				
25–34 years	3.9***	[3.1–5.0]	3.9***	[3.1–5.0]
35+ years	20.6***	[14.7–28.8]	20.6***	[14.7–28.7]
Marital status (Ref = Married)				
Not married	1.3	[0.9–2.1]	1.4	[0.9–2.1]
Education (Ref = Primary and below)				
Secondary and higher	0.7***	[0.6–0.8]	0.7***	[0.6–0.8]
Household SES (Ref = Non-poor)				
Poor	1.5***	[1.2–1.9]	1.5***	[1.2–1.9]

*p < 0.05; **p < 0.01; ***p < 0.001; Ref = reference category

## Discussion

The objective of this paper was to examine family planning use and fertility desires among women living with HIV who attended FP clinics in resource-limited settings in Kenya. The findings show that a high proportion of women in the sample were HIV-positive (13 %), which is much higher than the national rate of 5.6 % [38] .

### Family planning use and HIV status

We also found that a majority of women in the sample had used FP in the last 12 months and short-term contraceptives were the mostly commonly used methods. There were significant differences in FP use by HIV status. Similar to previous studies [30, 39], our study showed that HIV-positive women were more likely to use FP compared to HIV-negative women. However, over time, there was a general reduction in FP use and the decline was greater among HIV-positive than HIV-negative women. We could not find a plausible explanation for this finding. Similar to previous research [40], we found that most HIV-positive women used condoms compared to HIV-negative women. This finding suggests the FP advice received from providers is dominated by ‘condom’ messages. Further analysis, showed there was a slight increase in the average number of children desired among HIV-positive women at endline, which is consistent with the decline in FP use at endline. A previous study in Kenya and Zambia found no differences in the use of contraceptives between HIV-positive and HIV-negative women except in condom use [20]. Type of health facility was significantly associated with family planning use. Women who visited a health center had a higher likelihood of using family planning compared to women who went to a hospital. One plausible reason for this finding is that women only needed short-acting contraceptives and not long-acting contraceptives, which require a higher level of clinical skills and equipment than shorter-term methods and these, are more likely to be found in a higher-level facility.

Consistent with previous research conducted in Kenya to assess the attitudes toward family planning among HIV-positive pregnant women [35], our study findings showed that marital status was also an important determinant of family planning use. Women who were not married were less likely to use family planning compared to married women. Household socioeconomic status was associated with family planning use. Women from poor households were more likely to use family planning compared to their counterparts from wealthier households. This observation is further supported by the results from the multivariate regression model on fertility desires, which showed that women from poor households were more likely not to desire more children compared to women from wealthier households. One plausible explanation is that providers could be promoting more FP use to poorer households or (perhaps more likely) the motivation to use contraceptives is higher because of poverty or expense of bringing up children (poorer households feel the burden more). These findings are not consistent with previous research in Zambia and Swaziland which found that women from wealthier households were more likely to be associated with contraceptive use compared to women from poorer households [30].

### Dual protection and HIV status

A higher proportion of HIV-positive women were associated with dual method use compared to HIV-negative women. Previous Integra research also confirmed that dual method use was higher among HIV-positive than HIV-negative women in Swaziland [40]. Women who were not married were more likely to be associated with dual use of family planning compared to married women. This suggests that women who are not married are using condoms (most likely during casual sexual encounters) in addition to another family planning method unlike their married counterparts for whom condom use is low [38, 41, 42], and is mostly associated with disease and infidelity.

### Fertility desires and HIV status

The proportion of mistimed and unintended pregnancies was higher among HIV-positive women compared to HIV-negative women. This finding is similar to previous research, which found high numbers of mistimed pregnancies among Women living with HIV [39]. Among women with unintended pregnancy (regardless of HIV status), a majority reported using short-term family planning methods. This finding corroborates previous research conducted in Swaziland which found only short-acting methods were used prior to the most recent pregnancy [40]. This finding suggests that challenges still exist with regard to helping women avoid unintended pregnancies; further mixed-methods research with HIV-positive women reported elsewhere, suggests this may be because of lack of access to more effective (less user/partner dependent) long-acting contraception [43].

Similar to previous research [10, 21, 22, 29, 30, 43], our study findings show that at baseline, HIV-positive women were less likely to want more children compared to HIV-negative women. However, over time, there were no significant differences in fertility desires between the two groups. Age was a significant predictor of fertility desires. Generally, the likelihood of not wanting more children increased with age. This finding is consistent with previous studies which found that older women were less likely to want more children compared to younger women [21, 30]. Education level and household socioeconomic status were associated with fertility desires. Women with secondary and higher education were more likely to want more children compared to their counterparts with primary education and lower. Findings from previous studies on the association between education and fertility desires were mixed. In Zambia, women with no education and those with higher education were associated with a higher likelihood of wanting more children compared to those with secondary education. While in Lesotho, women with higher level education did not want to have more children (Johnson et al., 2009) compared to those with secondary education. However, a meta-analysis of fertility desires of people living with HIV found no strong association of fertility desire with better education (Berhan Y and Berhan A, 2013).

The findings from our study have important implications for policy and programmatic efforts aimed at addressing sexual and reproductive health and HIV needs of women. First, the proportion of women who are infected with HIV is higher than the national rate highlighting the need to ramp up efforts aimed at addressing the increased risk of acquiring HIV among women. Prevention of unintended pregnancies (i.e., for prevention of mother-to-child HIV transmission) and promotion of dual protection are important measures here. Second, our study results suggest that despite the high uptake family planning methods among HIV-positive women, the proportion of mistimed and unintended pregnancies was higher among HIV-positive women compared to HIV-negative women. This calls for need to strengthen family planning services for HIV-positive women by addressing the underlying factors that may place these women at increased risk of unintended pregnancies. At the global level, prevention of unintended pregnancies among HIV-positive women has been identified as one of the major components of a comprehensive strategy on prevention of mother-to-child (PMTCT) HIV transmission [19]. Our study found that short-acting contraceptives were the most commonly used methods. There is need to encourage the use of long-acting reversible contraceptive (LARC) to reduce the risk of unintended pregnancy. These methods do not require maintenance and last for a longer period compared to short-acting contraceptives.

The lost-to-follow-up (LFU) was 30 and 28 % for the intervention and comparison groups, respectively. The LFU was within the anticipated range (30 %) and a sensitivity check suggested that it was random not systematic, and, therefore, no effect on the findings.

### Limitations

Our study had a number of limitations that need to be highlighted. First, the data on HIV status was self-reported and, therefore, may have been subject to bias (either under reporting or over reporting, though the latter is unlikely) since individuals have a propensity to provide socially desirable responses, especially to questions on sensitive subject matter. Second, due to lack of data on cultural beliefs and practices, we were unable to assess the association between these factors and fertility desires among women living with HIV. Evidence shows that cultural factors play an important role in shaping decisions related to childbearing [21, 22]. Other Integra publications use qualitative data to explore cultural and stigma-related factors related to fertility and contraceptive use (Mayhew SH, Colombini M, Tomlin K, Kimani J, Warren C, Zhou W, Mutemwa R, for the Integra Initiative. I do not like getting a baby when I have not planned…” Fertility intentions, contraceptive practices and inappropriately met need, among women living with HIV in Kenya. (Submitted)), [44]. Also, since the data for this study was collected from women seeking family planning services from selected public health facilities in one of the eight administrative regions in Kenya, we cannot generalize about family planning use and fertility desires among all women in Kenya. However, our findings provide important insights about use of FP vis-à-vis unintended pregnancies among women living with HIV in resource-limited settings.

## Conclusion

In conclusion, our study findings highlight the need and importance of making family planning services an integral and continuous part of HIV prevention, treatment and care programming initiatives. HIV-positive women who desire to limit childbearing should be identified and supported to address factors exposing them to increased risk of unintended pregnancies.
